# The application of Compont gel in chronic obstructive jaundice rats model^1^

**DOI:** 10.1590/s0102-865020190050000004

**Published:** 2019-06-03

**Authors:** Wei-wei Luo, Xue-ling Zhou, Qing-qing Wang, Yan-jing Shao, Ze-ming Li, Dong-kang Zhao, Shui-ping Yu

**Affiliations:** I Master, Nephrology Department of Guilin NO. 924 Hospital, Guangxi Key Laboratory of Metabolic Diseases Research, Gulin Key Laboratory of Kidney Diseases Research, Guilin, Guangxi, China. Design of the study.; II PhD, Department of Hepatobiliary Surgery, the First Affiliated Hospital of Guangxi Medical University, Nanning, Guangxi, China. Scientific and intellectual content of this study.; III PhD, Department of Radiation and Medical Oncology, Zhongnan Hospital of Wuhan University, Wuhan, Hubei, China. Critical revision.; IV Master, Department of Clinical Laboratory of Guilin NO. 924 Hospital, Guangxi Key Laboratory of Metabolic Diseases Research, Gulin Key Laboratory of Kidney Diseases Research, Guilin, Guangxi, China. Technical procedures.; V Master, Department of General Surgery, The Affiliated Xingtai People’s Hospital of Hebei Medical University, Xingtai, Hebei, China. Technical procedures.; VI Master, Laboratory of Hepatobiliary and Pancreatic Surgery, Affiliated Hospital of Guilin Medical University, Guilin, Guangxi, China. Technical procedures.

**Keywords:** Jaundice Obstructive, Fibrosis, Liver, Rats

## Abstract

**Purpose:**

To establish a new rat model, the pathogenesis of which is closer to the clinical occurrence of chronic obstructive jaundice with liver fibrosis.

**Methods:**

90 SD rats were randomly divided into 3 groups. Group A common bile duct ligation, group B common bile duct injection compont and group C injection saline. The serum of three groups was extracted, and the liver function was detected by ELISA. HE staining, Masson staining and immunohistochemistry were used to detect liver pathology.

**Results:**

Group B showed a fluctuant development of jaundice, obstructive degree reached a peak at 2 weeks, and decreased from 3 weeks. HA, LA and PCIII were significantly higher than control group. 3 weeks after surgery, liver tissue fibrosis occurred in group B, and a wide range of fiber spacing was formed at 5 weeks. Immunohistochemistry showed that hepatic stellate cells were more active than the control group.

**Conclusion:**

Intra-biliary injection of Compont gel is different from the classic obstructive jaundice animal model caused by classic bile duct ligation, which can provide an ideal rat model of chronic obstructive jaundice with liver fibrosis.

## Introduction 

Obstructive jaundice is a kind of clinically common disease, mainly caused by cholestasis^1^. The bile outflow pathway is narrow or the bile can not enter the digestive tract after obstruction. It can cause pathophysiological disorders of the body, and eventually lead to intestinal flora imbalance and dysfunction, combined with severe septic shock and systemic multiple organ failure^2^. Obstructive jaundice often has an acute onset, rapid development, and high mortality^3^. The animal model of obstructive jaundice is an important part of the study of hepatobiliary diseases, and it is the basis for studying the pathogenesis of various hepatobiliary diseases, evaluating the therapeutic effect and drug development. Most of the existing obstructive jaundice animal models are constructed by bile duct ligation and biliary surgery^4^ ^,^ ^5^, which is not only complicated to operate, but has a lower survival rate of more than 3 weeks^6^. Moreover, the pathogenesis of obstructive jaundice caused by this method is quite different from that of clinical cholelithiasis. Clinically, the cause of obstruction, such as cholelithiasis, is characterized by gallstones or common bile duct stones. Under the action of dilated biliary tract and bile duct endothelial cells, stones enter and block the common bile duct and intrahepatic bile duct. The site of the disease is in the lumen, and the degree of obstruction is not completely blocked^7^ ^,^ ^8^. Animal models constructed by traditional bile duct ligation or biliary surgery cannot simulate this series of pathological processes, and the results may be affected. This study found that the injection of Compont gel in the common bile duct of rats can construct an animal model that is closer to the pathogenesis of clinical obstructive jaundice, and the survival rate is higher than 3 weeks. 

The main component of the medical Compont gel is a cyanoacrylate homologue with a small amount of stabilizer and polymerization inhibitor, which can be used for the closure of the skin surface near the surgical incision^9^ ^,^ ^10^. Clinically, Compnt glue is often used in surgery to stop bleeding, and hernia, dural repair^9^ ^,^ ^11^. The mechanism of the glue is that under the function of anions in the blood and tissue fluid of the wound, it can be rapidly polymerized and solidified into a film to produce an elastic thin film with high tensile strength^12^ ^,^ ^14^. Our preliminary experimental results suggest that the gel can quickly spread to the branches of the intrahepatic bile duct after injection into the common bile duct, forming acute obstruction within one week. Subsequently, it partially dissolves under the action of bile and bile duct to form incomplete obstruction, and the adherent film is distributed in each bile duct. Therefore, it is feasible to construct an animal model of chronic obstructive jaundice using the gel. 

This study aimed to find an animal model closer to the pathogenesis of clinical chronic obstructive jaundice, we used compont gel to inject the obstructive jaundice caused by the common bile duct in rats. The mechanism is closer to the pathogenesis of clinically common obstructive jaundice. This also provides a more practical rat model to better apply future basic experimental findings to clinical disease treatment. 

## Methods

### Groupings and models creation

 Rats were purchased from the Experimental Animal Center of Dongcheng Campus of Guilin Medical University. Compont gel was purchased from Beijing COMPONT company. There were three kinds of Compont gel specifications, which were 0.5ml/branch, 1ml/branch and 1.5ml/branch. The specification used in this experiment was 1ml/branch. 

Ninety female SD rats, weighing 180-200 g, were randomly divided into three groups, with 6 rats per period. Group A was the common bile duct ligation group. Group B was the Compont gel injection group that the common bile duct of rats was injected with Compont gel. Group C was the control group, in which rats’ common bile duct was injected with saline. Fasting for 8 hours before surgery, anesthesia was injected intraperitoneally with 1% barbital sodium, of which dose was 0.5 ml/100 g, and the anesthesia time was about 40 minutes. All the fur on the abdomen was cleaned and disinfected with 75% alcohol prior to surgery. The upper abdomen was opened by a median incision. Group A was ligated and disconnected from the common bile duct, group B was injected with 0.2 ml of Compont gel in the common bile duct, group C was injected with 0.2 ml of saline. The rat abdominal incision was sutured and placed in an incubator. 

### Detection of blood biochemistry and three items of liver fiber

 Venous blood was collected at 1, 2, 3, 4, and 5 weeks after surgery. After standing, the cells were centrifuged and separated. And the serum samples were stored in a refrigerator at -80°C for uniform detection. 

The blood biochemistry test was performed by the Department of Clinical Laboratory of the Affiliated Hospital of Guilin Medical University. The instrument was Backman AU5800. 

Three indicators of liver fiber were detected by enzyme-linked immunosorbent assay (ELISA), including rat hyaluronic acid (HA; kit was purchased from CUSABIO; No. CSB-E08120r), rat laminin (LN; kit was purchased from Elabscience; No. E-EL-R0039 C), and rat procollagen (PC III; kit was purchased from Elabscience; No. E-EL-R1096c). Perform the experimental procedure according to the instructions. First, equilibrate the various reagents to room temperature for 30 minutes. Each group is provided with a standard hole and a sample hole to be tested, and the reduplicate number of hole is 3. The sample and each gradient concentration of the test standard were sequentially added, 100 μl/well, 37℃ 2 hours. After the solution was dried, the biotin-labeled antibody working solution was added, 100 μl/well, 37℃ 1 hour. Then, the washing solution was added, 200 μl/well, repeating 3 times. Horseradish peroxidase-labeled avidin working solution was added, 100 μl/well, 37℃ 1 hour. After washing the plate 5 times, the substrate solution was added, 90 μl/well, shielded from light for 15-30 minutes at 37℃, and the stop solution was added, 50 μl/well. Finally, the optical density (OD value) at 450 nm was measured, and the concentration of the sample stock solution (serum) was calculated. 

### Tissue sections preparation

 Liver tissues of the left external lobe of the rats were excised at 1, 2, 3, 4, and 5 weeks after operation. The liver tissues were immersed in a 10% formalin solution for 48 hours. Subsequently, relying on the Department of Pathology of the Affiliated Hospital of Guilin Medical University, the paraffin block was embedded and tissue sections were prepared. 

### Hematoxylin eosin (HE) staining assay

 HE staining kit (Cat. no. G1120) was purchased from Solarbio company in Beijing. The specific steps are as follows: paraffin sections were deparaffinized with xylene (I) and xylene (II) for 5 min, then treated with a gradient of ethanol (anhydrous ethanol for 5 min, 95% 2 min, 80% 2 min, 70% 2 min), distilled water for 2 min; hematoxylin dyeing solution for 10 min, tap water washing; differentiation solution for 30 s; soaking in water for 15 min, then staining with eosin dyeing solution for 30 seconds, rinsing the sections under running water and immersing in water for 5 min. Alcohol gradient dehydration, xylene transparent, sealed with neutral gum. Finally, an optical microscope was used to observe and photograph. 

### Masson trichromatic staining assay

 Masson staining kit (Cat. no. G1340) was purchased from Solarbio company in Beijing. The experimental procedure was as follows: first, paraffin sections were dewaxed using xylene, and then Weigert iron hematoxylin staining solution was prepared and used for staining 5 min. The acidic ethanol differentiation solution was used for 5 s, and sections were washed with water. Use Masson blue solution to return to blue for 3 min and rinse with distilled water for 1 min. It was stained with Lichunhong Magenta staining solution for 5 min, and washed with a weak acid working solution (mixed solution of distilled water and weak acid solution in a ratio of 2:1) for 1 min. Continue to soak and clean the sections (phosphoric acid solution for 2 min, weak acid working solution for 1 min), then stain with aniline blue staining solution for 2 min, and weak acid working solution for 1 min. After 95% ethanol was rapidly dehydrated, it was dehydrated 3 times with absolute ethanol for 5 s/once. The xylene transparent, neutral gum was then used to seal the tablets. Finally, observe and take a picture using an optical microscope. 

### Liver Ishak scoring system ^*15*^

 Cirrhosis Ishak scoring system: In part A, the assessment is the hepatitis (fragmented necrosis) at the interface around or around the portal area. A score of 0 indicates no such change; a score of 1 represents mild (focal, a small number of portal areas); 2 points represent mild or moderate (focal, most of the portal area); 3 points represent moderate (continuity, <50) % of the door tube area and spacing area; 4 points represent serious (continuity, >50% of the door tube area and interval area). Part B, the assessment is confluent necrosis. 0 points means no such change; 1 point represents focal fusion necrosis; 2 points represent partial area 3 area necrosis; 3 points represent most area 3 area necrosis; 4 points represent 3 area necrosis occasional portal area - central vein (PC) bridging; 5 points represent 3 areas of necrotic multiple portal area-central vein (PC) bridging; 6 points represent total lobular or multiple lobular necrosis. Part C, assessment of focal (plaque) dissolved necrosis, apoptosis and focal inflammation. 0 points means no such change; 1 points means has 1 necrotic area or less per *10 times of field of view; 2 points means 2-4 necrotic areas per *10 times of field of view; 3 points means every *10 times of field of view There are 5-10 necrotic areas; 4 points represent more than 10 necrotic areas per *10 times of field of view. Part D, the assessment is the inflammation of the portal area. 0 points means no such change; 1 point means mild, part or all of the door tube area; 2 points means moderate, part or all of the door tube area; 3 points means moderate or severe, all door area; 4 points represent serious, all Door tube area. 

Fibrosis score: 0 points represent no fiber; 1 point represents fiber hyperplasia in some portal areas, with or without short fibrous septa; 2 points represent fiber hyperplasia in most portal areas, with or without short fibrous septa; 3 points represent most of the portal area There is fiber hyperplasia, occasionally the door tube area is fiber bridged; 4 points represent the fiber proliferation in the portal area, accompanied by obvious fiber bridging (between the door tube and the door tube and between the door tube and the central vein); 5 points represent Obvious bridge (door and door tube and / or door tube and central vein), occasionally nodules (not completely hardened); 6 points for possible or clear cirrhosis. 

### Immunohistochemistry assay

 Immunohistochemical kit (Cat. no. [E184]ab32575) was purchased from Abcam company in USA. The experimental protocol was as follows: the sections were deparaffinized twice by xylene, and hydrated by different concentrations of ethanol (anhydrous ethanol, 95%, 80%, 70%, distilled water). The antigen in the tissue was repaired by cooking in an autoclave with ethylenediaminetetraacetic acid (EDTA) buffer (pH = 8) for 3 min. Sections were then washed with phosphate buffered saline (PBS) and soaked in 3% hydrogen peroxide for 20 min to block endogenous peroxidase activity. Sections were incubated in 10% goat serum at room temperature for 30 min. Subsequently, α-SMA antibody (Cat. No. ab32575; Cambridge, MA, USA) was added to the slide, and placed in a wet box at 4 ℃ overnight. The dilution of α-SMA antibody is 1:500. Then rinse with PBS and incubate the secondary antibody for 1 hour at room temperature. Specimens were developed using 3-aminobenzidine stained hydrochloride (DAB). Use hematoxylin counterstaining, dehydration and neutral resin fixation. Finally, observe and take a picture using an optical microscope. 

The results were divided into four grades according to the degree of immunohistochemical staining: grade 0, <10% of cells stained positive; grade 1, 11%-25% of cells stained positive; grade 2, 26%-50% of cells Positive staining; grade 3, >50% cell staining positive. Immunohistochemical analysis and scoring were performed by two pathologists. 

### Statistical analysis

 All the data were analyzed using GraphPad Prism 5 (GraphPad Software Inc., San Diego, CA, USA) and SPSS18.0 (SPSS, Inc., Chicago, IL, USA). Serological indexes were presented as the mean ± standard deviation. Comparisons between two groups were conducted using the Student’s *t*-test, and *P*<0.05 was considered to indicate statistical significance.

## Results

### Three items of blood biochemistry and liver fiber

**Figure 1:**
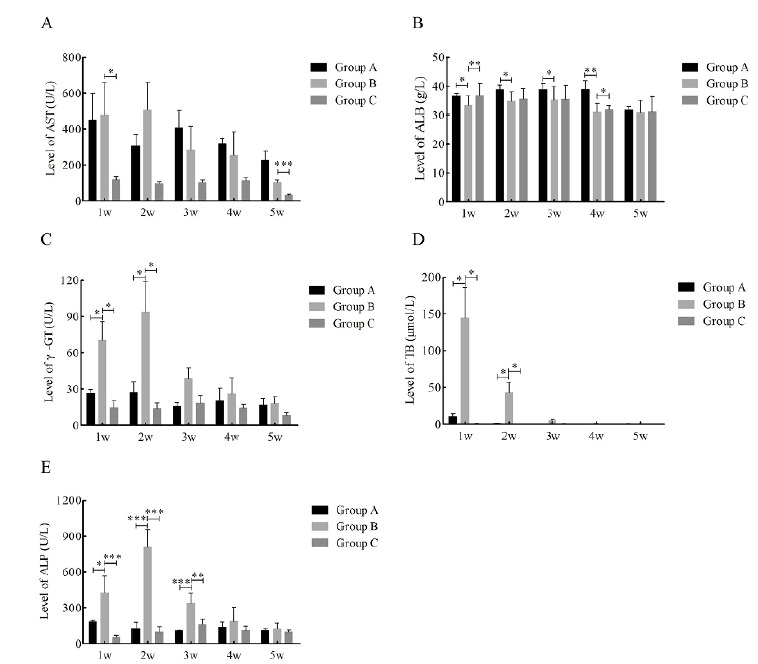
Changes in liver function from weeks 1 to 5 post-surgery. Changes of **A**, aspartate aminotransferase (AST); **B**, albumin (ALB); **C**, γ-glutamyl-transferase (γ-GT); **D**, total bilirubin (TB) and **E**, alkaline phosphatase (ALP). *, P<0.05; **, P<0.01; ***, P<0.001.

**Figure 2:**
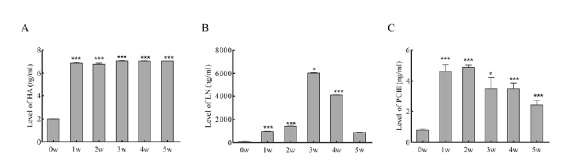
Three indexes of liver fiber in rat model. **A**, **B**, and **C** respectively represent the serum values of hyaluronic acids (HA), laminin (LN) and type III procollagen (PCIII). Statistical P values were obtained by comparing each experimental group with week 0. *, P<0.05; **, P<0.01; ***, P<0.001.

 After the rats were treated according to the experimental group, serum samples were collected at 1, 2, 3, 4, and 5 weeks, respectively. Blood biochemistry and liver fiber three indicators were detected by ELISA, and the statistics are shown in the chart (Table 1, Figs. 1 and 2). 

**Table 1:** Blood biochemical index of rat model. The mean ± standard deviation (mean ± SD) was used to indicate the values of each serum, n=6. Group A was compared with group B, aP <0.05, bP <0.01. Group B was compared with group C, cP <0.05, dP <0.01.

Group	AST	ALB	ALP	TB	γ-GT
1 w					
Group A	449.00±150.64	36.60±0.90^a^	183.67±10.07^a^	10.63±4.26^a^	26.33±3.22^a^
Group B	478.33±182.17^c^	33.30±3.30^d^	424.55±141.65^d^	42.88±14.38^c^	70.67±15.14^c^
Group C	118.00±18.68	36.73±4.25	50.67±17.89	0.80±0.62	14.67±5.86
2 w					
Group A	306.67±63.69	38.83±1.63^a^	125.33±54.24^b^	1.10±0.70^a^	27.00±9.17^a^
Group B	506.83±154.12	34.80±3.23	806.25±147.08^d^	40.87±14.39^c^	93.67±25.15^c^
Group C	95.33±13.87	35.67±3.51	99.67±41.76	0.50±0.20	14.00±4.58
3 w					
Group A	406.00±98.80	38.90±1.97^a^	107.67±2.52^b^	0.20±0.10	15.67±3.22
Group B	285.50±131.53	35.42±4.51	337.20±85.57^d^	4.80±1.77	38.50±9.03
Group C	103.33±14.05	35.60±4.77	162.33±44.23	0.53±0.31	18.33±6.43
4 w					
Group A	318.67±30.09	39.93±3.06^b^	136.33±45.79	0.27±0.15	20.33±10.21
Group B	257.83±127.60	31.08±3.01^c^	186.91±115.83	0.60±0.42	26.00±13.01
Group C	113.67±17.79	32.03±1.21	108.67±36.69	0.27±0.21	14.33±3.22
5 w					
Group A	225.67±51.62	31.90±1.05	108.67±17.01	0.80±0.10	17.00±5.29
Group B	105.75±10.94^d^	30.98±4.27	123.00±51.28	0.45±0.30	18.25±5.56
Group C	34.00±5.29	31.20±5.21	99.33±12.90	0.17±0.11	8.00±2.65

Compared with group C, the levels of aspartate aminotransferase (AST), γ-glutamyl-transferase (γ-GT), total bilirubin (TB) and alkaline phosphatase (ALP) in group B began to increase at 1 week after surgery, peaked at 2 weeks (AST 506.83±154.12 U/L; γ-GT 93.67±25.15 U/L; TB 42.88±14.38 μmol/L; ALP 806.25±147.08 U/L), decreased at 3 weeks after surgery, and became normal at 5 weeks, which was statistically significant. Compared with group A, the trend of these four blood biochemical indicators was roughly the same as before. In addition, the level of ALB decreased at 1 week postoperatively and was statistically significant compared with group C. 

The levels of hyaluronic acid (HA), laminin (LN) and type III procollagen (PCIII) in group B are shown in the Figure 2. Compared with preoperative, the level of HA increased significantly from the postoperative period, and reached the peak at 3 weeks. The level of LN gradually increased from the postoperative period, and peaked at 3 weeks, and decreased at the 4 weeks after surgery. The level of PCIII increased significantly from the postoperative period, peaked at 2 weeks, and then began to decrease. (On 3 weeks, HA, *P*=0.008; LN, *P*=0.04; PC III, *P*=0.04). 

### General situation

**Figure 3:**
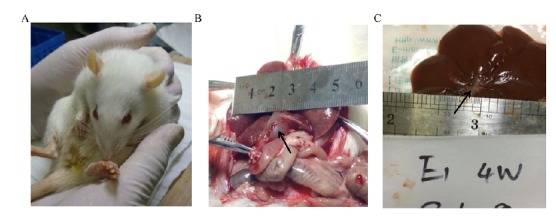
General observations of group B rats (Injecting Compont into the common bile duct). **A**, the representative image of rats with notable yellowing of skin; **B**, the representative image of rats with expanded and thinned common bile duct on 1 week after operation (black arrow indicates expansion of the common bile duct); **C**, the representative image of rats with restored form of common bile duct on 4 week after operation (black arrow indicates restored of the common bile duct).

 On the third day after surgery, compared with the C group, the rats in the A group and the B group had poor mental state, poor diet, and yellow staining on the skin, ears, and urethra (Fig. 3A). One week after surgery, it was observed that the common bile duct of rats in group A and group B showed an expansion of about 1.0 cm in diameter, and the bile duct wall became thin and spherical (Fig. 3B). Subsequently, our re-observation at the 4th week after surgery, it was found that the degree of bile duct dilation was reduced, the diameter of the common bile duct was narrowed to 0.4 cm, and the wall of the tube was significantly thickened (Fig. 3C). 

### The observation result of HE staining light microscope

**Figure 4:**
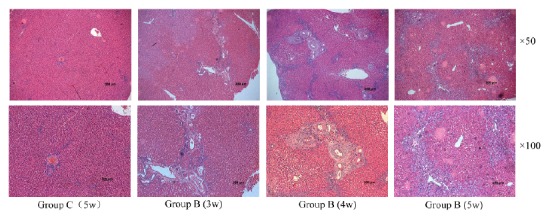
The liver tissue of rats was examined by H-E staining assay. Upper HE×50 and lower ×100, from left to right C group (bile duct injection of normal saline for 5 weeks), group B (bile duct injection compont glue) for 3, 4, 5 weeks. The shape of the C group was normal, the bile duct epithelial injury and the fine bile duct reacted in group B, the fibrous hyperplasia around the bile duct and the irregular mass of the clad hepatocytes.

 According to different groups, the rats were treated with different surgical conditions. From 1 week after operation, the liver tissue was taken weekly and fixed to prepare paraffin blocks until the end of the 5th week. Tissue sections were treated by HE staining (Fig. 4). Observed by light microscopy (magnification ×50), from the third week after surgery, compared with the negative control group (group C), the experimental group (group B) presented bile duct epithelial lesions, including bile ducts, increased number of epithelial cells, morphological disorders, thickening of the basement membrane; and thin bile duct reactions, such as choledochal interstitial edema, increased number of small bile ducts, bile duct dilatation, etc.; and fibrosis, wrapping hepatocytes to form irregular masses, and over time. As the fiber membrane is thickened to fiber spacing, fibrosis gradually increases. Until the fifth week, the fibrous septum was completely wrapped around the central sinus. A clearer image was observed using a 100× optical microscope. In addition, the negative control group (group C), 5 weeks after surgery, HE staining showed that the morphology of the hepatic sinus and central sinus was basically normal. According to the Ishak score^15^, the control group had a part A score of 0, a part B score of 0, a part C score of 1, and a part D score of 1, totaling 2 points. The experimental group had a part A score of 1 at 3 weeks, a part B score of 0, a part C score of 1, and a part D score of 2 at a total of 4 points. The experimental group had a part A score of 2 at 4 weeks, a part B score of 1, a part C score of 1, and a part D score of 2, for a total of 6 points. The experimental group had a part A score of 2 at 5 weeks, a part B score of 1, a part C score of 1, and a part D score of 3, for a total of 7 points.

### The observation result of Masson staining light microscope

**Figure 5:**
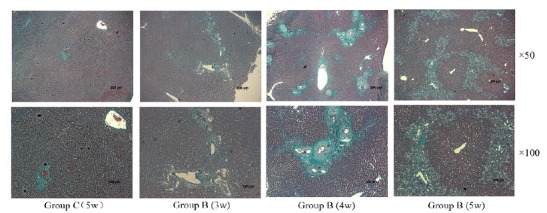
The liver tissue of rats was examined by Masson staining assay. Upper Masson×50 and lower ×100, from left to right C group (bile duct injection of normal saline for 5 weeks), group B (bile duct injection compont glue) for 3, 4, 5 weeks. Group C had normal structure. In group B, the number of small bile ducts increased, and gradually became flaky. The proliferation of collagen fibers around the bile duct, thickening of the bile duct wall and proliferation of the fibers extended to the normal hepatic cord, and the hepatocytes were packed into clusters. The severity of the lesion was positively correlated with the postoperative time.

 The method of preparing liver tissue paraffin sections was the same as the HE staining test, and then liver tissue sections were subjected to collagen staining (Fig. 5). Observed by light microscopy (magnification ×50), it was found that the fiber around the portal vein gradually increased (blue), the number of small bile ducts increased, the collagen fibers around the bile duct proliferated, the bile duct wall thickened, and the fibrous septum and small nodules gradually formed. With time, the fibrosis was thick, and the area affected by the liver expanded, until the fifth week, the fibrous septum wrapped liver tissue to form irregular nodules. Using a 100× mirror, the above morphological changes were more clearly visible, that was, the bile duct wall fibers gradually thickened, the fibers around the lumen increased, and the proliferative fibers extended to the normal hepatic cord and surround the hepatocytes to form nodules. In contrast, 5 weeks after surgery, in the negative control group (group C), the morphology of the hepatic sinus and central sinus was basically normal, with only a small amount of wall fibers and fiber-free membrane. Finally, according to the Ishak fibrosis scoring system^15^, we can conclude that the control group is 0 points, the experimental group is 2 points at 3 weeks, the experimental group is 4 points at 4 weeks, and the experimental group is 5 at 5 weeks. 

### Biliary tube morphology after Compont injection

**Figure 6:**
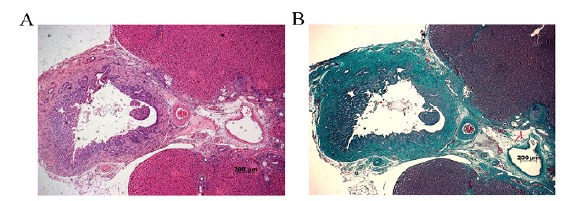
Representative image of biliary tube morphology after Compont injection. HE and Masson×50, there were no obvious damages in the bile duct epithelial cells and adjacent hepatocytes. Scale bar 200 μm.

 At the 3rd week after operation, rat liver tissue was taken and paraffin sections were prepared. The morphology of common bile duct was observed by HE staining and Masson staining (Fig. 6). We found that after compont surgical gel injection, the common bile duct wall thickened, but not with bile duct epithelial cells and hepatocyte damage, and no large-scale apoptosis and necrosis. Therefore, we conclude that Compont surgical glue does not damage the bile duct and liver. In addition, we guess that compont surgical glue-induced bile duct obstruction is not associated with cholangiocarcinoma and hepatotoxicity.

### The results of immunohistochemical staining

**Figure 7:**
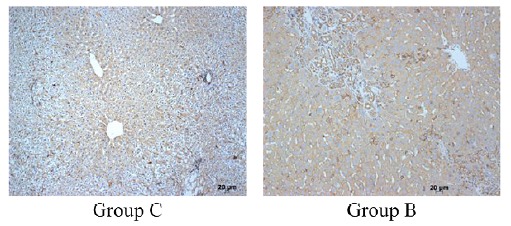
Representative image of liver tissue after immunohistochemical staining. The group C (bile duct injection of saline) for 5 weeks, the B group (bile duct injection compoint glue) for 5 weeks. Scale bar 20 μm.

 Hepatic stellate cell (HSC) are central cells in the development and transformation of liver fibrosis^16^. In liver injury, HSC activation is converted to myofibroblast (MFB), which produces a number of growth factors that stimulate extracellular matrix (ECM) deposition through autocrine and paracrine signaling pathways, such as α-Smooth muscle actin (α-SMA), which ultimately promotes ECM deposition^17^ ^,^ ^18^; At the same time, α-SMA can be used as a marker protein for HSC proliferation activation. In addition, during the development of liver fibrosis, PDGF-A and PDGF receptors are formed in the early stage of MFB proliferation and ECM production^19^. PDGF-A overexpression can further induce HSC differentiation and liver fibrosis. Thus, α-SMA is closely related to ECM and can be used to detect the activity level of HSC. We took the liver tissue of rats at the 5th week after surgery and used immunohistochemistry to detect the expression of α-SMA (Fig. 7). It was found that in the negative control group (group C), α-SMA was mainly expressed in the hepatic cord and nucleus, and only a small amount was expressed in the cytoplasm. In contrast, the α-SMA of the experimental group (group B) was abundant in the hepatic cytoplasm, completely filled the entire cell, and no expression in the nucleus. Endoscopic score, the experimental group was 3 (more than 50% of positive cells), the control group was 1 (less than 25% in positive cells).

## Discussion

The classic bile duct ligation method covers almost all biliary models, including obstructive jaundice models, bile internal and external drainage models, and so on^4^ ^,^ ^5^. It has a wide range of applications, but the model principle of this classic procedure is to rely on external force to block the biliary tract, which is different from the pathogenesis of clinical biliary diseases. There are many kinds of diseases that can cause obstructive jaundice in clinical practice. Only a few are complete obstruction of biliary tract caused by tumor compression. Most of them are cholelithiasis and chronic biliary obstruction. Their pathogenesis is that obstruction directly blocks biliary tract or biliary tract stenosis. Ulcers and scars formed by detachment of bile duct epithelial cells can aggravate biliary stricture. The rupture and relaxation of muscle fibers of the wall can lead to biliary dilatation, suggesting that biliary endothelial cells are closely related to biliary stricture. Therefore, we speculate that clinical biliary occlusion or compression stenosis, its pathogenesis may involve the role of biliary endothelial cells and wall muscles. Obviously, the ligation model of the classical method does not fully apply these changes. In addition, biliary obstruction caused by clinical diseases is often chronic development, and the course of disease is repeated. The biliary tract alternates with obstruction and recanalization under the intervention of the body itself or medical measures. Except for congenital biliary atresia, there are few diseases with complete obstruction. Thus, the classic common bile duct ligation does not seem to be applicable to all studies of biliary diseases, especially chronic obstructive biliary diseases. Therefore, exploring animal models that are closer to the pathogenesis of clinical chronic obstructive jaundice has potential application value. 

Biliary stent is the most direct and effective method to relieve biliary obstruction. It is widely used in biliary and pancreatic diseases^20^ ^,^ ^21^, and orthotopic liver transplantation^22^ ^-^ ^24^. It is important for survival after orthotopic liver transplantation. However, biliary tract and pancreatic stents have a certain rate of recurrent obstruction^25^, and the specific mechanism of function is still unclear. Considering that there is a certain correlation between the material of the biliary stent^26^ ^-^ ^28^ and the individual differences of the patient, the function of the biliary muscle layer and the endothelium may also be a factor that cannot be ignored. Therefore, the production of a model for biliary epithelial research has potential clinical value. The successful production of this type of model can provide long-term obstruction and a large area of compressed bile duct. 

Through experimental research, we found that Compont gel is injected into the common bile duct and spreads rapidly and coagulates. It can effectively obstruct for more than 5 weeks, providing longer obstruction channels, obstruction time and larger area of endothelial cell compression. Perhaps it can be used for the study of biliary stents and biliary endothelium. This study has limited the study of the relationship between biliary epithelium and long-term compression for a variety of reasons. After the administration of Compont gel in the common bile duct of rats, yellow staining was observed in the rats 3 days after operation, and the dilated common bile duct was seen in the open abdomen, and AST, γ-GT, TB and ALP were significantly increased. Peaks appeared at 2 weeks, and decreased at 3 weeks after surgery. The level of ALB was decreased. These results suggest that Compont gel blocks the common bile duct and causes obstructive jaundice in rats, but the degree of obstruction is relieved at 3 weeks after surgery. Three indicators of liver fibrosis were detected. The results showed that the levels of HA, LN and PCIII were increased, and the trend was roughly the same as the blood biochemical indicators, that is, the indicators began to decrease at 3 weeks after surgery. In addition, the results of HE staining and Masson staining of liver tissue sections showed that the rats in the common bile duct were injected with Compont gel, and the bile duct reaction appeared from the third week after operation, and the fiber interval package appeared at the 5th week after operation. Irregular nodules formed by liver tissue. At the same time, the results of immunohistochemistry indicated that α-SMA was expressed in the liver cytoplasm of the experimental group, which was closely related to EMC deposition and liver fibrosis. Based on the results of this part of the experiment, we speculated that Compont gel can cause obstructive jaundice in rats. At 3 weeks after operation, Compont gel partially dissolved under the action of bile, causing incomplete obstruction and forming a bile duct stent structure. In order to rule out the toxic side effects of Compont gel on the common bile duct, we added the pathology of the common bile duct after injection of this bio-adhesive. After consultation with the pathologist, it was concluded that the thickening of the common bile duct wall did not show any damage, apoptosis or necrosis of the bile duct epithelial cells or hepatocytes.

We found that Compont gel was injected in the biliary tract at a dose of 0.2 ml. Immediately after the injection, it formed a complete obstruction and then gradually dissolved, resulting in incomplete obstruction lasting more than 5 weeks. Compared with the common bile duct ligation, the survival rate of the Compont gel injection group was higher, 93% (28/30), and 2 rats died in the first week of acute obstruction. Serum indicators and pathological sections showed that the rats with liver fibrosis were 25/30, and the model success rate was 83%. In addition, the most significant difference from the common bile duct ligation is that the injection method does not affect the integrity of the bile duct wall, and there is almost no direct damage to the bile duct endothelial cells and wall function. This model can be used for the study of the physiological functions of the bile duct endothelium and the wall muscles after chronic obstructive jaundice and biliary stent placement. 

## Conclusions

The obstructive jaundice model produced by our group, Compont gel can contact bile duct without damaging the bile duct endothelium, and then can form incomplete obstruction, and the obstruction time is longer, which is more effective to simulate the pathogenesis of clinically common diseases that cause obstructive jaundice. The chronic obstructive jaundice model formed by injection of Compont gel can be used for the study of chronic biliary tract disease, stent stenosis and biliary tract injury, and provides a more ideal animal model for clinical disease research. 
